# Mechanisms of Chinese Medicine Xinmailong’s protection against heart failure in pressure-overloaded mice and cultured cardiomyocytes

**DOI:** 10.1038/srep42843

**Published:** 2017-02-16

**Authors:** Jianyong Qi, Juan Yu, Yafang Tan, Renshan Chen, Wen Xu, Yanfen Chen, Jun Lu, Qin Liu, Jiashin Wu, Weiwang Gu, Minzhou Zhang

**Affiliations:** 1 AMI Key Laboratory of Chinese Medicine, Guangdong Province Academy of Chinese Medicine, Guangdong Province Hospital of Chinese Medicine, 2nd Affiliated Hospital of Guangzhou University of Chinese Medicine, Guangzhou 510006, China; 2 Animal Laboratory, Southern Medical University, Guangzhou, 510515, China; 3 Animal Laboratory, 2nd Affiliated Hospital of Guangzhou University of Chinese Medicine, Guangzhou 510006, China; 4 Lab of Chinese Materia Medica Preparation, 2nd Affiliated Hospital of Guangzhou University of Chinese Medicine, Guangzhou 510006, China; 5 Puning Hospital of Chinese Medicine, Puning, Guangdong Province, 515300, China; 6 Department of Pharmaceutical Sciences, College of Pharmacy, Northeast Ohio Medical University, Rootstown, Ohio, 44272, USA

## Abstract

Patients with heart failure (HF) have high mortality and mobility. Xinmailong (XML) injection, a Chinese Medicine, is clinically effective in treating HF. However, the mechanism of XML’s effectiveness on HF was unclear, and thus, was the target of the present study. We created a mouse model of pressure-overload-induced HF with transverse aortic constriction (TAC) surgery and compared among 4 study groups: SHAM (n = 10), TAC (n = 12), MET (metoprolol, positive drug treatment, n = 7) and XML (XML treatment, n = 14). Dynamic changes in cardiac structure and function were evaluated with echocardiography *in vivo*. In addition, H9C2 rat cardiomyocytes were cultured *in vitro* and the phosphorylation of ERK1/2, AKT, GSK3β and protein expression of GATA4 in nucleus were detected with Western blot experiment. The results showed that XML reduced diastolic thickness of left ventricular posterior wall, increased ejection fraction and fraction shortening, so as to inhibit HF at 2 weeks after TAC. Moreover, XML inhibited the phosphorylation of ERK1/2, AKT and GSK3β, subsequently inhibiting protein expression of GATA4 in nucleus (*P* < 0.001). Together, our data demonstrated that XML inhibited the TAC-induced HF via inactivating the ERK1/2, AKT/GSK3β, and GATA4 signaling pathway.

Heart failure (HF) impacts approximately five million Americans and is listed in 11% of all death certificates (284, 388) in the United States in 2013^1^, and is also becoming one of the most prevalent heart diseases in Asia, including in China^2^. HF is associated with a damaged structure and/or function of the left ventricle, leading to impaired left ventricular ejection fraction (LVEF). It has been widely accepted that the impairment of LVEF may be caused by acute myocardial infarction and associated with aging, diabetes, obesity, etc. Although it has been studied for more than one century, HF remains the worst disease without an effective cure. The high mortality and non-availability of suitable drug treatments led to persistent interests and efforts in developing effective therapies and suitable preventive measures for HF patients at an early stage in the hope of improving their life span and quality^3^.

Xinmailong (XML), a bioactive composite extracted from periplaneta Americana (Fig. 1a), has been widely used in China to treat patients with HF^4^,^5^. As shown in Fig. 1b–e, XML contains 4 active ingredients: adenosine, inosine, protocatechuic acid, and pyroglutamate dipeptides. A recent clinical trial in HF patients confirmed the effectiveness of XML in improving cardiac function and reducing complications along with few adverse effects^6^.

Although it is clinically effective in treating HF patients, the underlying mechanisms of XML’s action on HF remain elusive. The current study evaluated a hypothesis that XML can inhibit cardiac remodeling, thus, protect heart from HF. To evaluate this hypothesis, we examined a mouse HF model with XML pretreatment and compared the effects of XML with control groups.

## Methods

### Animals and Reagents

This study was performed in accordance with the guidelines and with approval from the Institutional Animal Care and Use Committee of Guangdong Province Hospital of Chinese Medicine, Guangzhou University of Traditional Chinese Medicine, and with the Guide for the Care and Use of Laboratory Animals published by the National Academy of Sciences (8^th^ edition, Washington DC, 2011). Wild-type C57BL/6 J mice (10–12 weeks old male, 25 ± 5 g body weight) were obtained from the Experimental Animal Center of Guangdong Province. Formalin (10% neutral buffered) was purchased from WEX Corp (Guangzhou, China). Pentobarbital sodium was purchased from Sigma-Aldrich Corp (Guangzhou, China).

### Preparation of XML

**Figure 1:**
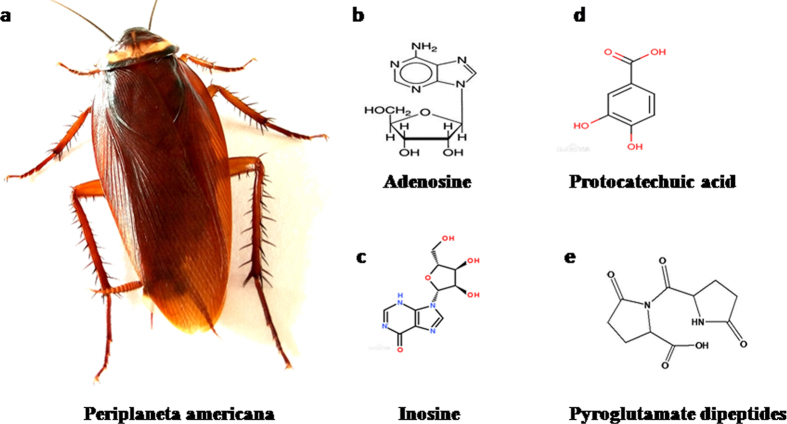
XML is extracted from Periplaneta Americana (**a**). The main active components of XML include adenosine (**b**), inosine (**c**), protocatechuic acid (**d**), and pyroglutamate dipeptides (**e**).

XML were provided by Yunnan Tengchong Pharmaceutical Corporation (Yunnan, China, batch: 130272). Each milliliter XML contains 12–18 mg composite nucleosides and bases, 16–24 mg combined amino acids, 1.0–1.6 mg uracil (C4H 4N2O2), 1.7–2.5 mg hypoxanthine (C5H5N4O), and 3.5–5.0 mg inosine (C10H12N4O5). Main active components of XML are adenosine, inosine, protocatechuic acid, and pyroglutamate dipeptides (Fig. 1b–e), as determined with following methods: ① Detection of composite nucleosides and bases: XML stock was diluted 1:1,000 in 0.05 M glycine HCl buffer (PH 3.0) and then examined with ultraviolet-visible spectrophotometry (SOP-QC06304, UV wavelength: 254 nm). Contents were calculated as follows: C = (A/490) × 10 × n, where C is the content of composite nucleosides and bases per 1 ml XML, A is the absorbance of the test solution, 490 is the average absorbance value at 254 nm when the mixing concentration of composite nucleosides and bases was 1%, and n is the dilution ratio. ② Detection of combined amino acids: (1) Preparation of calibration curve. Ten mg Glutamate was dissolved in 200 ml warm water. Solution of 0.2, 0.4, 0.6, 0.8, and 1.0 ml were transferred into test tubes, and then diluted to 1 ml with water. Each test tube was added with 1.0 ml citrate buffer (0.2 mol/L, pH 5.0) and 1.0 ml ninhydrin test solution, heated in water bath for 15 minutes, and then cooled immediately. Three ml 60% ethanol were added to each test tube 5–10 minutes later. The above solutions, with corresponding reagent as blank control, were examined with the spectrophotometer at 570 nm. (2) Preparation of XML test solution. Stock solution was diluted 50 times with water, then 1 ml diluted solution was pipetted into an ampoule and also into a 10 ml volumetric flask. One ml hydrochloric acid was added to the ampoule, which was then sealed, hydrolyzed at 110 °C for 8 hrs, and then cooled and dried. The residue was dissolved in 10 ml water, and its total amino acids contents were determined. Water was added into the 10 ml volumetric flask to the scale, and the free amino acids contents were determined. (3) Measurement. Absorbency of the above two test solutions and water were measured separately using the same method as ‘Preparation of Calibration curve’. The content of combined amino acids was calculated by subtracting free amino acids from total amino acids. ③ Detection of Uracil, Hypoxanthine and Inosine: (1) Experiments of chromatogram conditions and system suitability. Octadecyl silane boned silica gel was used as a filler, 0.05 mol/L acetate buffer (pH 5.0) as the mobile phase A, methanol-0.05 mol/L acetate buffer (pH 5.0) (80:20) as the mobile phase B. The gradient elution was started with a flow rate of 0.5 ml/min (Spectrophotometer at 254 nm). The number of theory plates was not less than 3000 as determined by the peaks of Uracil. (2) Preparation of reference solution. Uracil (30 μg), Hypoxanthine (40 μg) and Inosine (70 μg) and mobile phase A were added to each ml solution. (3) Preparation of test solution. XML stock (1 ml) was diluted into 50 ml mobile phase A. (4) Measurement. Reference and test solutions (10 μl each) were injected separately.

### Protocol

Based on literature, clinical usage (5 mg/kg/day for adults), and the Meeh-Rubner equation of dose conversion, the human equivalent dosage of XML is 45.5 mg/kg/day for mouse. We choose 50 mg/kg dosage for mice by intraperitoneal injection (i.p.) daily. Mice were assigned to one of the four groups: SHAM, TAC, MET, and XML. Mice in SHAM received saline i.p. and all surgery except aorta constriction; mice in TAC were subjected to saline i.p. and TAC surgery; MET mice received metoprolol i.p. and TAC surgery; XML mice received XML i.p. and TAC surgery.

### Transverse Aortic Constriction (TAC)

The method to impose pressure overload in mice was described previously^7^. In brief, mice were anesthetized with pentobarbital sodium (60 mg/kg) and mechanically ventilated. Aortic constriction was created via a center thoracotomy by ligation of the transverse thoracic aorta between the innominate artery and left common carotid artery with a 28-gauge needle using a 7–0 braided polyester suture. Sham operation was performed without aortic constriction.

### Echocardiography

Before euthanasia, *in vivo* left ventricular (LV) function and LV hypertrophy were assessed by measuring fraction shortening (FS) and left ventricular diastolic posterior wall thickness (LVPWd) with echocardiography using a Vevo 770 echocardiography system (Visual Sonics, Toronto, Canada) with a 30 MHz linear array transducer^8^. Briefly, Animals were anesthetized with isoflurane/oxygen inhalation, once the short-axis two-dimensional (2D) image of the left ventricle was obtained at the papillary muscle level, 2D guided M-mode images crossing the anterior and posterior walls were recorded. Diastolic thickness of left ventricular posterior wall (LVPWd) and inner dimension of diastolic or systolic left ventricles (LVIDd and LVIDs) were measured in M-mode. Following parameters were calculated: Fraction shortening (FS) = (LVIDd–LVIDs)/LVIDd, end-diastolic volume (EDV) = ((7.0/(2.4 + LVIDd)) × LVIDd^3^, and end-systolic volume (ESV) = ((7.0/(2.4 + LVIDs)) × LVIDs^3^.

Doppler echocardiography was used to assess pressure gradient across the constriction and evaluate the degree of stenosis in the aortic banding studies^9^. A no imaging Doppler pencil transducer (continuous wave) was placed at the apex and orientated towards the proximal ascending aorta. The peak velocity (in meters per second) was measured, and the maximum instantaneous gradient (millimeters of Hg) was calculated using the Bernoulli equation: aortic pressure gradient (AoPg) = 4 × (velocity)^2^.

### Heart weight assessment and histological examination

At the completion of experiment, animals were euthanized and their hearts were removed. Body weights (BW) and heart [left ventricle + right ventricle] weights (HW) were determined. The left ventricle was quickly separated from the atria and right ventricular free wall and fixed in 4% paraformaldehyde overnight before embedding in paraffin. Sections of 7.5 μm were prepared and stained with hematoxylin-eosin (HE) or Sirius red for evaluations of myocyte hypertrophy and collagen content, respectively.

Mean values of cardiomyocytes in the HE-stained LV cross sections from each mouse were calculated from 60 to 80 cells using light microscopy at 400× magnification. Sirius-stained sections were quantitatively analyzed using a light microscope at 40× magnification and a color image analyzer (QWinColour Binary 1, LEICA) to evaluate myocardial fibrosis (red fibrotic area as opposed to yellow myocardium).

### Cell Culture

Rat H9C2 cardiomyocytes cells (American Type Culture Collection, Manassas, VA, USA) were maintained in DMEM supplemented with 10% fetal calf serum at 37 °C in CO_2_ incubation. The medium was replaced every 2–3 days. Cells were sub-cultured or subjected to experimental procedures at 80–90% confluence. Cells were preincubated with 10 μm metoprolol to block β-adrenergic receptors or with XML (0.75 mg/ml) for 30 min before stimulation with 1 μm isoproterenol (ISO).

### Western blot analysis

The proteins of H9C2 cells were extracted with RIPA Lysis Buffer (Beyotime, China) containing 1% Phenylmethanesulfonyl fluoride on ice. Protein concentration was detected with bicinchoninic acid assay kits. We mixed 30 μg proteins with 5 × SDS-PAGE sample loading buffer. Following 5 min heating at 95 °C, denatured proteins were subjected to 10% Tris-glycine gel and transferred electrophoretically to polyvinylidene difluoride membranes (Bio-Rad). Then 5% bovine serum albumin in TBST (Tris-buffered saline, 0.1% Tween 20) was used to block non-specific sites at room temperature for 1 h. The membranes were incubated with the primary antibodies overnight at 4 °C and washed with TBST three times. Secondary horseradish peroxidase-conjugated antibodies (1:10,000) were incubated with the membranes for 1 h at 37 °C. Antibody-positive bands were visualized using a VersaDoc imaging system (Bio-rad, USA). Data were analyzed by using Quantity One (Bio-rad, USA). GAPDH and eIF5 (Santa Cruz Technology, Delaware, CA, USA) were used as loading controls. Following antibodies were used: anti-phospho-ERK1/2 (Thr202/Tyr204), anti-phospho-PKB (Ser473), anti-phospho-p38 (Thr180/Tyr182), anti-phospho-JNK (Thr3/Tyr185), anti-p38, anti-JNK, and anti- PKB (Cell Signaling Technology, Beverly, MA, USA), anti-ERK1/2, anti-GSK3b and anti-phospho (p)-GSK3b (Ser9), anti-GAPDH and anti-eIF-5 (Santa Cruz Technology, Delaware, CA, USA). Peroxidase and bands were visualized using a super western sensitivity enhanced-chemiluminescence detection system (ECL kit, Pierce Biotechnology, Pierce, IL, USA). Autoradiographs were quantitated with a densitometry Science Imaging System (Bio-Rad, Hercules, CA, USA).

### Cell viability assay

The effect of XML on cellular proliferation and viability was determined by 3-(4,5-dimsethylthiazol-2-yl)-2,5-diphenyl tetrazolium bromide (MTT) assay (R&D Systems, UK) and bromodeoxyuridine (BrdU) ELISA assay (Mannheim, Germany). H9C2 cells were seeded in 96-well plates at a density of 3.0 × 10^3^ cells/well in 100 μl medium and allowed to attach overnight. The cells were then treated with XML at increasing concentrations (0–5 mg/ml) for 24, 48, and 72 h. Subsequently, the cells were incubated with MTT and BrdU reagents at a final concentration of 5 mg/ml for 4 h. Finally, the intracellular formazan crystals were solubilized with 150 μl DMSO. Absorbance was measured at 490 nm using an enzyme-linked immunosorbent assay plate reader, and the reduction in cell viability in treatment groups was expressed as the percentage compared with the XML-treated and XML-free control cells. All experiments were performed in triplicate.

### Statistical analysis

Data are presented as mean ± S.E.M. Statistical analysis was performed by one-way analysis of variance followed by Turkey’s method or unpaired two-tailed Student’s t-tests. Results were considered statistically significant at p < 0.05.

## Results

### XML enhanced cardiac function following TAC (echocardiography)

To explore the effects of XML on HF *in vivo*, we produced a HF model by TAC surgery and monitored cardiac function by echocardiography. As shown in Table 1, heart rates (HR) were between 460–490 bpm (without significant differences among the four groups, *P* > 0.05), suggesting that the mice were near physiological condition (500~650 bpm). The AoPg was 40–60 mmHg in the TAC, MET and XML mice (significantly higher than SHAM group, *P* < 0.001), leading to HF after 2 weeks due to pressure overload. These results demonstrated that the TAC models were successful and stable. In 2 weeks, LVPWd was significantly increased in the TAC group (LVPWd, TAC, 1.02 ± 0.03 mm vs. SHAM, 0.718 ± 0.02 mm, *P* < 0.001), while decreased in MET and XML groups (LVPWd, MET, 0.812 ± 0.03 mm vs. XML, 0.860 ± 0.02 mm, *P* < 0.01), revealing that XML could inhibit the wall thickening in the HF model. Moreover, FS was significantly reduced in TAC mice (SHAM, 45.41 ± 2.45% vs. TAC, 28.32 ± 2.54%, *P* < 0.001), suggesting occurrence of HF in the TAC group. EF also reduced in the TAC group (SHAM, 80.98 ± 1.72% vs. TAC, 59.97 ± 4.96%, *P* < 0.001, Table 1), further supporting the occurrence of HF in TAC mice. However, both EF and FS were increased in the MET and XML groups (*P* < 0.05), indicating that XML could reverse cardiac remodeling in structure and systolic function.

**Figure 2:**
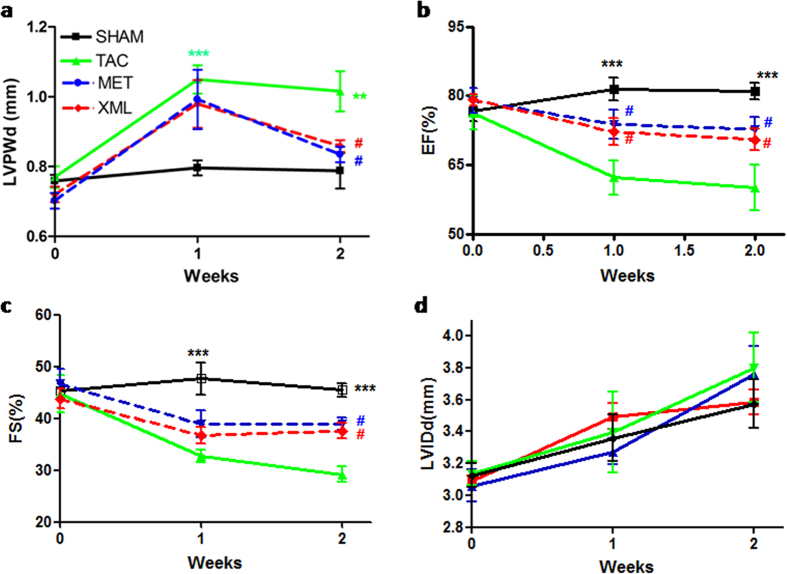
Dynamic changes in cardiac structure and function were detected by echocardiography in the SHAM, TAC, XML, and MET groups. (**a**) LVPWd (**b**) EF (**c**) FS and (**d**) LVIDd were observed dynamically after 0, 1, and 2 weeks. **P < 0.01 and ***P < 0.001 indicate significant differences from SHAM. ^**#**^*P* < 0.05 indicates significant difference from TAC group.

We monitored dynamical changes in echo. As shown in Fig. 2, LVPWd of all 3 groups (TAC, MET, XML) increased in 1 week compared with SHAM (*P* < 0.001, Fig. 2a). At the end of 2 weeks, LVPWd in TAC group reduced slightly, suggesting a thinner wall thickness and forming of decompensate cardiac hypertrophy. Also, EF and FS were reduced after 2 week with slower reduction in the XML and MET than in TAC mice (*P* < 0.05). However, there were no significant differences in LVIDd among the 4 groups (*P* > 0.05, Fig. 2d). Together, these date demonstrated that XML could alleviate heart failure in response to pressure overload.

### XML inhibited heart failure after TAC (histology)

To further characterize the effects of XML on HF, we analyzed cardiac histology with HE/Sirius staining. As shown in Table 2, HWs were significantly higher in the TAC group than SHAM group (TAC, 180.8 ± 6.84 mg vs. SHAM, 118.4 ± 3.79 mg, *P* < 0.001), while no significant differences in body weight among the four groups (*P* > 0.05). The ratio of left ventricular weight (LVW) to tibal length (TL) was increased in TAC mice compared with SHAM mice (TAC, 8.34 ± 0.49 vs. SHAM, 4.90 ± 0.20, P < 0.01). Moreover, lung weight and the ration of lung weight to tibial length (Lung/TL) were significantly increased in TAC mice (Lung: 134.2 ± 3.92 mg vs. 193.5 ± 21.2 mg, *P* < 0.05; Lung/TL: 7.73 ± 0.20 vs. 11.0 ± 1.27, *P* <* *0.05; SHAM vs. TAC, respectively), suggesting formation of lung edema in TAC mice. However, there were no significant differences in liver weight and in the ratio of liver to TL between SHAM and TAC groups (*P* > 0.05). Together, these results further demonstrated that HF was formed after TAC surgery.

**Figure 3:**
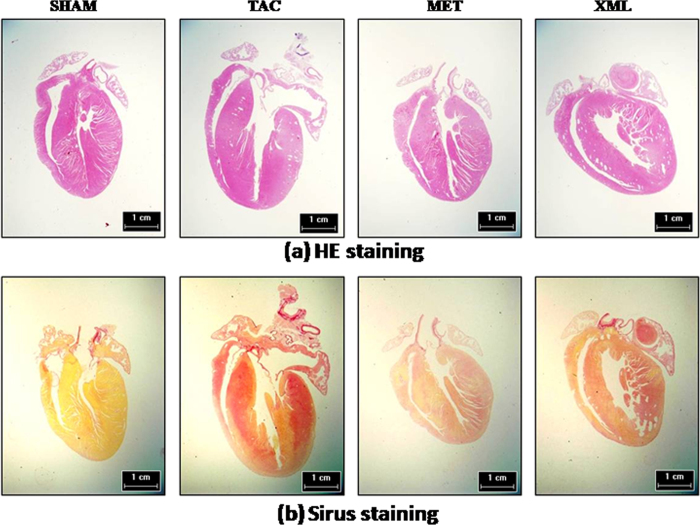
Anatomical data in SHAM, TAC, MET, and XML mice. (**a**) HW, (**b**) the ratio of LV weight (LW) to tibial length (TL), (**c**) the ratio of lung weight to TL, and (**d**) the ratio of liver weight to TL were compared. *P < 0.05, **P < 0.01, ***P < 0.001 indicate significant difference. n.s.: no significant difference.

**Figure 4:**
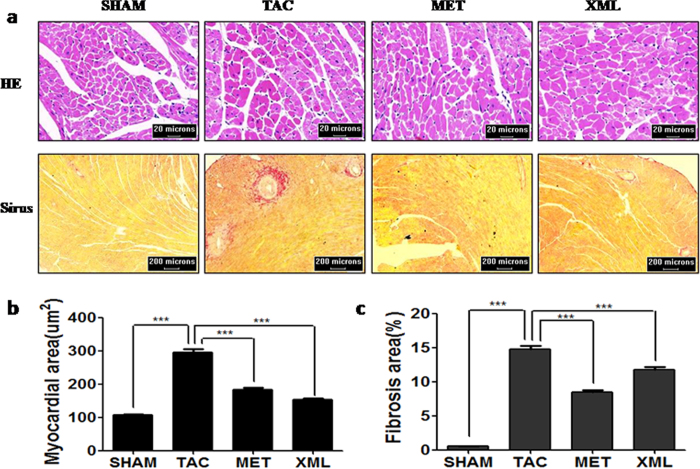
Dye-stained HF heart sections. (**a**) H&E-stained (upper) and (**b**) Sirius red-stained (lower) sections of representative hearts from SHAM, TAC, MET and XML mice. Scale at bottom is in cm.

**Figure 5:**
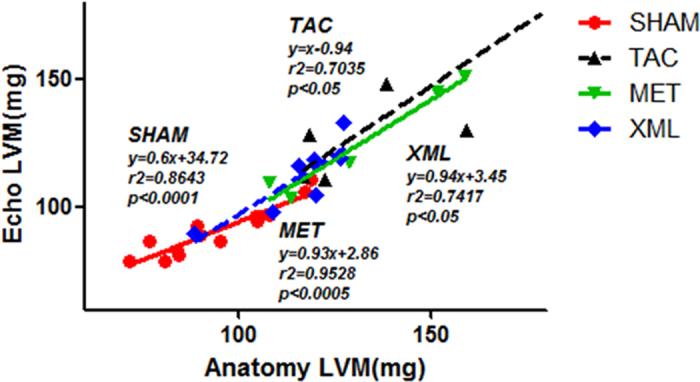
Positive linear correlation between the weights of echocardiographic left ventricular myocardium (Echo LVM) and anatomical LVM in four models (SHAM, TAC, MET, and XML mice).

We compared the effects among TAC, MET and XML mice. HW and LVW/TL were significantly reduced in MET and XML mice (MET: HW, 151.9 ± 1.21 mg, LVW/TL, 6.391 ± 0.37; XML: HW, 150.8 ± 7.96 mg, LVW/TL, 6.655 ± 0.21, respectively) compared with TAC mice (HW, 180.8 ± 6.84 mg, LVW/TL, 8.34 ± 0.49, *P* < 0.01). At the end of 2 weeks, cardiomyocytes were much bigger in TAC than SHAM mice (TAC, 295.1 ± 10.42 μm vs. SHAM, 106.8 ± 2.61 μm, *P* < 0.001, Figs 3a and 4a,b), but smaller in MET and XML mice (184.1 ± 5.56 μm vs. 152.6 ± 3.73 μm, *P* < 0.001). Cardiac fibrosis was also formed in TAC mice (14.76 ± 0.51% vs. 0.55 ± 0.02%, *P* < 0.001, Figs 3b and 4a,c), and reduced in MET and XML mice (MET: 8.88 ± 0.28%, XML: 7.22 ± 0.26%, *P* < 0.001). In addition, there was a positive linear correlation between the weights of echocardiographic left ventricular myocardium (Echo LVM) and anatomical LVM among the 3 groups (Fig. 5). In all, these data demonstrated that XML could inhibit HF in response to pressure overload.

### Phophsrylations of ERK1/2 and AKT after TAC surgery were inhibited by XML

**Figure 6:**
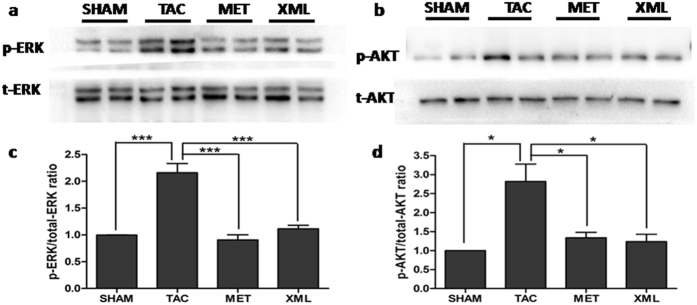
XML inhibited the phosphorylation of ERK1/2 and AKT after pressure overload *in vivo*. (**a** and **b**) Phosphorylated (p)-Thr202/204 extracellular signal regulated kinase (ERK) 1/2. (**c** and **d**) p-Ser473 protein kinase (**b**) (AKT). Data (mean ± SEM, *n* = 3) were expressed as fold changes from phosphorylated and total protein (ERK1/2, AKT). *P < 0.05, **P < 0.01.

To investigate the potential mechanisms of the inhibitive effects of XML on pressure-overload-induced HF, we focused on MAPK and AKT, two main signal transduction pathways involved in HF^10^. Using Western-blot analyses, we found that the ERK1/2 phophsrylations of threonines at 202^th^ and 204^th^ sites were increased in TAC group, compared with SHAM group (*P* < 0.05, Fig. 6a and c). Interestingly, the phosphorylations of threonines at 202th and 204th sites were reduced after XML stimulation, compared with TAC group (*P* < 0.001). Thus, XML reversed the ERK1/2 phosphorylationsof threonines at 202^th^ and 204^th^ sites. Moreover, the AKT phosphorylation of tyrosine at 473^th^ site was increased in TAC group (*P* < 0.05, vs. SHAM), but decreased after XML stimulation. Together, these data revealed that XML inhibited the phosphorylations of ERK1/2 and AKT in response to pressure overload *in vivo*.

### Phophsrylation of ERK1/2 and AKT/GSK3β were reduced after XML stimulation in rat H9C2 cardiomyocytes

**Figure 7:**
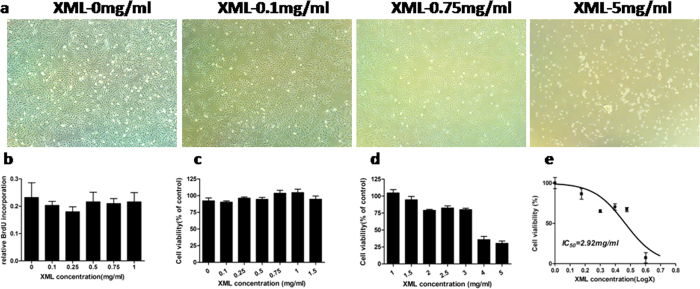
XML inhibited the proliferation of H9C2 cell line *in vitro*. (**a**) microscopic photography, (**b**) BrdU and (**c**,**d**) MTT assays were detected in different XML concentrations from 0.1 to 5 g/ml. (**e**) Dose-dependent curve was obtained to normalize the inhibition effect of XML on H9C2, IC50 = 2.92 mg/ml.

To determine dose response of XML in rat H9C2 cardiomyocytes, we evaluated the viability of H9C2 cells by microscopic photography and BrdU and MTT assays. As shown in Fig. 7a, proliferation of H9C2 cardiomyocytes were not influenced by 0.1, and 0.75 mg/ml XML, while significantly inhibited by 5 mg/ml XML. BrdU and MTT (Fig. 7b,c) assays showed absence of toxicity from 0.1 to 1.5 mg/ml XML. Over 1.5 mg/ml, XML inhibited H9C2 cell proliferation dose-dependently (Fig. 7d) and displayed moderate cytotoxicity with an IC50 of 2.92 mg/ml (MTT assay, Fig. 7e). Therefore, we chose 0.75 mg/ml as the final concentration of XML for H9C2 cell treatment.

**Figure 8:**
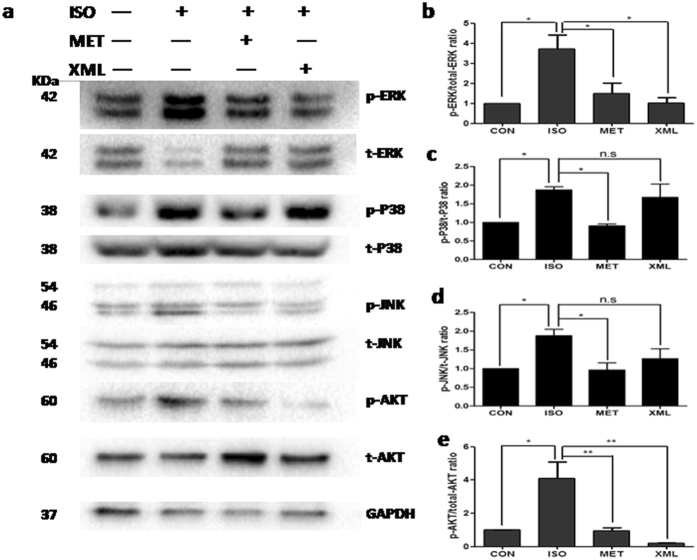
XML inhibited the phosphorylation of ERK1/2, and AKT after ISO stimulation. (**a**) Western blotting was performed to detect the phosphor-ERK1/2, JNK, p38, and AKT. Total proteins and GAPDH were used as loading control. (**b**) p-ERK1/2, (**c**) p-P38, (**d**) p-JNK, and (**e**) p-AKT (mean ± SEM, *n* = 3) were expressed as fold changes from phosphorylated and total proteins. *P < 0.05, **P < 0.01, n.s.: no significant difference.

**Figure 9:**
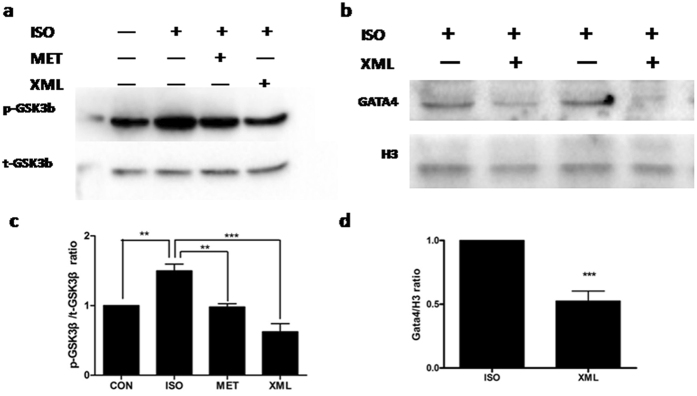
XML inhibited the phosphorylation of GSK3β and nucleus protein expression of GATA4. (**a** and **c**) Phosphorylated (p)- GSK3β and (**b** and **d**) nucleus GATA4. Data (mean ± SEM, *n* = 3) were expressed as fold changes from GSK3β phosphorylated/total protein and GATA3/H3. **P < 0.01, ***P < 0.001.

To simulate the pressure-overload model *in vitro*, we pre-incubated rat H9C2 cells with 1 μM isopronolol for 10 minutes. Western blot experiments showed similar results in the *in vitro* cardiomyocytes as in the *in vivo* study. The phosphorylation of ERK1/2 was increased by ISO stimulation but blocked after XML stimulation (*P* < 0.05, Figs 8a and 9b). Also, AKT phosphorylation of tyrosine at 473th site was decreased after XML stimulation (*P* < 0.01, Fig. 8a and e). Furthermore, we detected p38 and JNK of the MAPK family. There was no change in the phosphorylation of p48 and JNK after XML stimulation (Fig. 8a,c and d). Together, these data further confirmed the observation that XML reversed the ERK1/2 phosphorylations of threonines at 202th and tyrosine at 204th sites, and the AKT phosphorylation of tyrosine at 473th site *in vivo* and *in vitro*.

Furthermore, we detected a down-stream target of AKT, GSK3β. We found that the phosphorylation of GSK3β were increased after ISO pre-incubation and inhibited by XML (*P* < 0.001, Fig. 9a and c). Together, these data revealed that XML inhibited the ERK1/2 phosphorylations of threonines at 202^th^ and 204^th^ sites and AKT/GSK3β in response to ISO stimulation.

### The Expression of GATA4 was reduced after XML stimulation

To further investigate potential roles of the ERK1/2 and AKT pathways in inhibiting HF by XML, we analyzed a down-stream target, GATA4, which is a zinc finger containing transcription factor that plays key roles in promoting heart growth and regulating cardiac hypertrophy and heart failure^11^,^12^, and is associated with multiple hypertrophic signaling pathways, such as ERK1/2^13^, p38^14^, AKT^15^, and CnA/NFATc3^16^. We separated nuclear GATA4 protein from cytoplasm proteins. The expression of GATA4 in nuclear was significantly lower in the XML group than ISO stimulation group (*P* < 0.001), which revealed that XML inhibited the nuclear protein expression of GATA4 after ISO stimulation (Fig. 9b and d).

**Figure 10:**
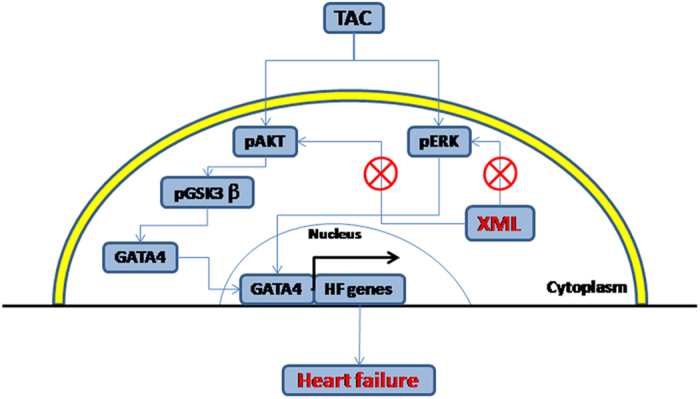
A model of pathways in the cardio-protection of XML in response to pressure stress overload. TAC (stress overload) could activate phosphorylation of the protein kinases of ERK1/2, AKT and GSK3β, enhance the expression of GATA4, promote the transcription of HF gene, and result in heart failure. XML could inhibit the phosphorylation of ERK1/2 and AKT, decreasing the expression of GSK3β and GATA4, leading to inhibit heart failure. ^⊗^Denotes inhibition of protein kinase by XML treatment.

Based on the above observations, we formulated following working model (Fig. 10): Pressure overload (TAC) activates MAPK and AKT/GSK3β, and subsequently promotes GATA4 trans-location from cytoplasm to nucleus, activates HF genes, and finally leads to HF; XML inhibits the phosphorylation of ERK1/2, and AKT/GSK3β pathway, which subsequently decreases the expression of transcription factor GATA4, resulting in inhibition of heart failure.

## Discussion

We constructed a HF model by TAC surgery as a platform to evaluating the effects of XML on HF. Our results showed that although cardiac systolic function (LVFE and FS) was reduced in the HF model along with pulmonary edema, these pressure-overload-induced decreases in cardiac systolic function were reversed by XML stimulation via a mechanism involving the ERK1/2, AKT/GSK3β and GATA4 signaling pathways.

### Pressure overload induced HF

Many animal HF models have been developed and widely studied, including TAC, myocardial infarction, and rapid pacing, etc. Among these procedures, TAC is one of the most common mouse models to induce cardiac hypertrophy and HF *in vivo*^17^,^18^,^19^. After TAC, heart mass increases initially (compensated hypertrophy) to normalize wall stress and maintain cardiac output. However, if the TAC stimulus is sufficiently intense or prolonged, the ventricles dilate, leading to diminished cardiac function and heart failure (decompensated hypertrophy). In the present work, we used a 28 g needle to construct the TAC model. As shown in Fig. 2A, AoPg was stable within 40~60 mmHg in TAC. The elevated AoPg caused HF after 2 weeks of TAC, in consistence with literature^20^.

The transition from compensated hypertrophy to HF involves various molecular and cellular events, e.g.: (1) myocyte hypertrophy; (2) increased expression of fetal gene programs and decreased expression of adult gene programs; (3) excitation–contraction decoupling and reduced contraction; (4) myocyte necrosis or apoptosis; and (5) changes in the extracellular matrix. Finally, these events lead to pump impairment. In the current study, we created the TAC pressure overload-HF mouse model, assessed the echo- and electrocardiographic indicators, fibrosis, and brain natriuretic peptide (BNP) levels, detected dynamic changes in cardiac structure and function with echo, and validated and correlated the changes with anatomic data (Fig. 7).

HF involves a network of signaling pathways, such as β-adrenergic receptor signaling and associated kinases, PKCα, Ca^2+^ /calmodulin-dependent kinase II signaling, Phosphodiesterase 5, MAPKs, HDAC, PI3-K/AKT pathways, *et al*.^12^ MAPK and AKT signaling pathways are critical in the response of cardiomyocytes to pressure overload^13^. Especially, AKT has been shown to promote cardiac hypertrophy. Shiojima *et al*. reported that prolonged and augmented AKT activation exacerbated compensatory hypertrophy to HF, mainly due to an unbalance in cardiomyocyte hypertrophy to angiogenesis^21^. Our study found that XML reduced the AKT activation by ISO stimulation. The similar response patterns between ERK and AKT signaling suggests a compensating mechanism of the two pro-hypertrophic kinases, in consistency with the previous report^7^.

### XML Treatment for Heart Failure

XML has been widely used to treat HF in clinics and hospitals for nearly 10 years in China. Liu *et al*. assessed the effects of XML injection in clinical HF patients for 3 months^4^ using Gated Myocardial Perfusion Imaging (G-MPI) to evaluate left ventricular ejection fraction (LVEF), left ventricular end-diastolic volume (LVEDV) and left ventricular end-systolic volume (LVESV). They found that LVEDV, LVESV, and plasma BNP levels were significantly lower, while LVEF was significantly higher in the XML group than in the control group (all P < 0.05). Ma *et al*. compared the effects of XML with control treatment in clinical HF patients for 15 days and found that XML significantly improved angiotensin II (AngII), high sensitivity C-reactive protein (hsCRP), pro-B-type natriuretic peptide (NT_proBNP), Left Ventricular End Systolic Volume Index (LVESVI), and left ventricular ejection fraction (LVEF)^5^.

In addition, XML was reported to reduce myocardial expression of hypoxia-induced factor-1α (HIF-1α) and decrease plasma endothelin-1 (ET-1) levels, so as to alleviate hypoxia-ischemic myocardial injury^22^. Also, it was reported that XML had therapeutic effects on early kidney damage induced by toxin of grass carp bile^23^. Our study experimentally confirmed XML’s anti-HF effect and supported XML’s clinical value in improving cardiac function of HF patients.

### GATA4 and HF

GATA4 is a key regulator and transcriptional factor of cardiac hypertrophy and HF, mediating gene expressions in response to hypertrophic/HF stimuli, e.g., pressure overload, isoproterenol, phenylephrine, via activating a variety of HF-associated genes, such as atrial natriuretic factor (ANF), BNP, and β-myosin heavy chain (β-MHC)^24^. GATA4 is also activated by MAP kinase and AKT signaling pathways, as confirmed in the current study (Figs 5 and 6). Recent studies investigated the roles of GATA4 in hypertrophy and HF. For example, over-expression of GATA4 by adenoviral gene transfer induced hypertrophy in cultured cardiomyocytes. Expression of dominant negative GATA4 or antisense GATA4 mRNA blocked the induction of GATA4-directed transcriptional responses and features of cardiomyocyte hypertrophy by phenylephrine and endothelin-1 in culture^25^. Mild trans-generic over-expression of GATA4 in mouse heart induced progressive hypertrophic responses *in vivo*^26^. GATA4 is also negative regulated by glycogen synthase kinase, which could reduce both basal and isoproterenol-induced nuclear expression of GATA4 and suppress GATA4 transcriptional activity^27^.

The current study showed that β-adrenergic stimulation by ISO increased ERK1/2/GATA4 complex in rat H9C2 cardiac myocytes. Interestingly, XML reduced GATA4 expression in nucleus. Therefore, the current study suggested GATA4 signaling is an important pathway in XML’s protection against HF.

### Study limitation

Although this study shed light on the cellular and molecular mechanisms of the anti-HF effect of XML, the complex mechanisms and multiple signaling pathways are still far away from fully known, in part due to the complex profile of active ingredients in XML, which is a general feature of Chinese Medicine. XML was also shown to inhibit multiple signaling molecules (e.g., Calcineurin, CamKII, data not shown) of ISO stimulation in H9C2 cardiomyocytes *in vitro*^7^.

Our HF animal model was created with 4-weeks TAC. We showed that the progression of HF in our model recapitulated many aspects of human HF, such as pulmonary edema, increased HW, LVW, LVW/TL, etc. Similar TAC mouse model was also used in Sadoshima’ lab^28^. They reported reductions of cardiac systolic function (EF and FS) following significant increases in AoPg to >40 mmHg after 2 weeks TAC surgery. However, short-term, high strength pressure-overload could also induce HF *in vivo* and could have different mechanisms as the TAC-induced HF.

## Conclusion

In conclusion, this study provided evidences that XML protected against induction of HF by pressure-overload via inhibiting both ERK1/2 and AKT dependent signaling pathways. Understanding the molecular mechanisms of XML on HF, particularly identifying the protective roles of XML on β-AR signaling in cardiomyocytes, is of critical importance to improve treatment of patients with heart failure. In this regard, this study provided experimental evidences to support XML as an effective therapy to treat patients with heart failure.

## Additional Information

**How to cite this article:** Qi, J. *et al*. Mechanisms of Chinese Medicine Xinmailong’s protection against heart failure in pressure-overloaded mice and cultured cardiomyocytes. *Sci. Rep.* **7**, 42843; doi: 10.1038/srep42843 (2017).

**Publisher's note:** Springer Nature remains neutral with regard to jurisdictional claims in published maps and institutional affiliations.

## Figures and Tables

**Table 1:** Echocardiological data.

	SHAM	TAC	MET	XML
HR	481.5 ± 10.3	484.4 ± 21.1	465.8 ± 12.0	483.5 ± 13.1
AoPg	4.703 ± 0.88	51.90 ± 7.12^***^	43.03 ± 8.13^**^	41.16 ± 5.42^**^
LVAW;d (mm)	0.718 ± 0.02	1.019 ± 0.03^***^	0.863 ± 0.05^**#**^	0.822 ± 0.05^**##**^
LVAW;s (mm)	1.352 ± 0.06	1.494 ± 0.09	1.413 ± 0.07	1.390 ± 0.10
LVID;d (mm)	3.576 ± 0.06	3.708 ± 0.21	3.668 ± 0.22	3.638 ± 0.16
LVID;s (mm)	2.085 ± 0.12	2.424 ± 0.29	2.453 ± 0.36	2.442 ± 0.29
LVPW;d (mm)	0.715 ± 0.02	1.017 ± 0.06^***^	0.860 ± 0.03^**#**^	0.812 ± 0.02^**##**^
LVPW;s (mm)	1.352 ± 0.06	1.494 ± 0.09	1.413 ± 0.07	1.390 ± 0.10
EF (%)	80.98 ± 1.72	59.97 ± 4.96^***^	72.70 ± 2.61^**#**^	71.33 ± 1.74^**#**^
FS (%)	45.41 ± 2.45	28.32 ± 2.54^***^	37.78 ± 1.08^**##**^	40.52 ± 2.29^**##**^
LV Mass (mg)	95.04 ± 3.99	140.6 ± 9.68^***^	115.1 ± 3.76^**#**^	116.5 ± 4.95^**#**^
LV Vol;d (ul)	53.87 ± 2.09	59.57 ± 8.16	57.97 ± 8.44	56.69 ± 6.06
LV Vol;s (ul)	15.20 ± 2.13	22.72 ± 6.21	23.64 ± 8.48	23.66 ± 6.30

Echocardiographic data of the four models (SHAM, TAC, MET, and XML mice). LV, left ventricle; TAC, transverse aortic constriction; MET, metoprolol, XML, Xinmailong injection, HR, heart rate; AoPg, aortic pressure gradient; LVAWd, LV end-diastolic anterior wall thickness; LVAWs, LV end-systolic anterior wall thickness; LVIDd, inner dimension of diastolic LV; LVIDs, inner dimension of systolic LV; LVPWd, LV end-diastolic posterior wall thickness; LVPWs, LV end-systolic posterior wall thickness; EF, eject fraction; FS, fractional shortening; LVM, mass of LV; LVMc, corrected mass of LV; LVVd, LV end-diastolic volume; LVVs, LV end-systolic volume. ***P < 0.001 compared to SHAM of the corresponding group. ^**#**^*P* < 0.05, ^**##**^*P* < 0.01 compared to TAC of the corresponding MET, XML groups.

**Table 2:** Anatomical data from study from study.

	SHAM	TAC	MET	XML
Number (n)	10	12	7	14
BW (g)	21.00 ± 0.30	20.17 ± 0.24	20.57 ± 0.37	20.36 ± 0.31
HW (mg)	118.4 ± 3.79	180.8 ± 6.84^***^	151.9 ± 1.21^**#**^	150.8 ± 7.96^**##**^
LVW (mg)	84.73 ± 3.30	144.0 ± 8.54^***^	110.9 ± 6.11^**#**^	115.9 ± 3.40^**#**^
Lung (mg)	134.2 ± 3.92	193.5 ± 21.2^*^	164.5 ± 6.68	168.4 ± 13.0
Liver (mg)	1077. ± 40.8	1169. ± 23.5	1168. ± 30.0	1064. ± 52.6
TL (mm)	1.735 ± 0.01	1.731 ± 0.01	1.729 ± 0.01	1.734 ± 0.01
HW/TL	6.829 ± 0.23	10.39 ± 0.41^***^	8.618 ± 0.10^**#**^	8.722 ± 0.47^**#**^
LVW/TL	4.900 ± 0.20	8.342 ± 0.49^***^	6.391 ± 0.37^#^	6.655 ± 0.21^**##**^
Lung/TL	7.734 ± 0.20	11.00 ± 1.27^*^	9.751 ± 0.77	9.484 ± 0.38
Liver/TL	62.01 ± 2.07	67.56 ± 1.31	67.51 ± 1.59	61.40 ± 3.12

Anatomical data of the four models (SHAM, TAC, MET, and XML mice).

BW: body weight; HW: heart weight; LVW: LV weight; TL: tibial length; ***P < 0.001, comparison of TAC with corresponding SHAM group; ^**#**^*P* < 0.05 and ^**##**^*P* < 0.01, comparisons of TAC with corresponding MET or XML group.
